# 274. *Legionella bozemanii* (*Fluoribacter bozemanae*) Brain Abscess in a Renal Transplant Recipient

**DOI:** 10.1093/ofid/ofab466.476

**Published:** 2021-12-04

**Authors:** Abrar Khan, Sreechandra L Kruthiventi, Rahul Mahapatra, Soma Sanyal, Wesley Kufel

**Affiliations:** 1 SUNY Upstate University, Jamesville, New York; 2 Upstate Medical University, Syracuse, New York; 3 State University of New York Upstate, Syracuse, New York; 4 Binghamton University, Binghamton, New York

## Abstract

**Background:**

Legionnaires’ disease is a potentially fatal multi-system disease caused by *Legionella* species. However, extra-pulmonary *Legionella* disease is rare and is typically associated with Legionella species other than *L. pneumophila* in immunocompromised patients.

**Methods:**

We present a 55-year-old immunocompromised male with history of living-related renal transplant secondary to IgA nephropathy (day 0) which was complicated by T-cell mediated rejection requiring anti-thymocyte globulin and elotuzumab (day 130).

**Results:**

Patient was hospitalized on day 184 with community-acquired pneumonia and treated with piperacillin-tazobactam and azithromycin. Three weeks later (day 214), he presented with new-onset seizures and was found to have a frontal brain abscess on MRI. His clinical course and brain imaging worsened despite undergoing multiple operative drainage procedures, placement of an extra ventricular drain, and receiving broad-spectrum antimicrobials. *L. bozemanii* was first identified from cerebrospinal fluid (CSF) on buffered charcoal yeast extract (BCYE) agar from day 240 and was also later confirmed by 16S rRNA sequencing. Susceptibilities were unavailable due to poor organism growth. Of note, his allergy history was significant for rash with ciprofloxacin and levofloxacin. Based on the low severity of the allergic reaction and need for central nervous system penetration, moxifloxacin 400 mg intravenously every 24 hours was initiated on day 244 in addition to broad-spectrum antibiotics. Subsequent CSF cultures were positive for *L. bozemanii* until the CSF culture on day 250. Due to poor clinical response, azithromycin and intrathecal polymyxin B were added for salvage therapy on day 255. His neurological status continued to worsen and he eventually succumbed to his illness on day 262.

08/31/20 MRI Brain

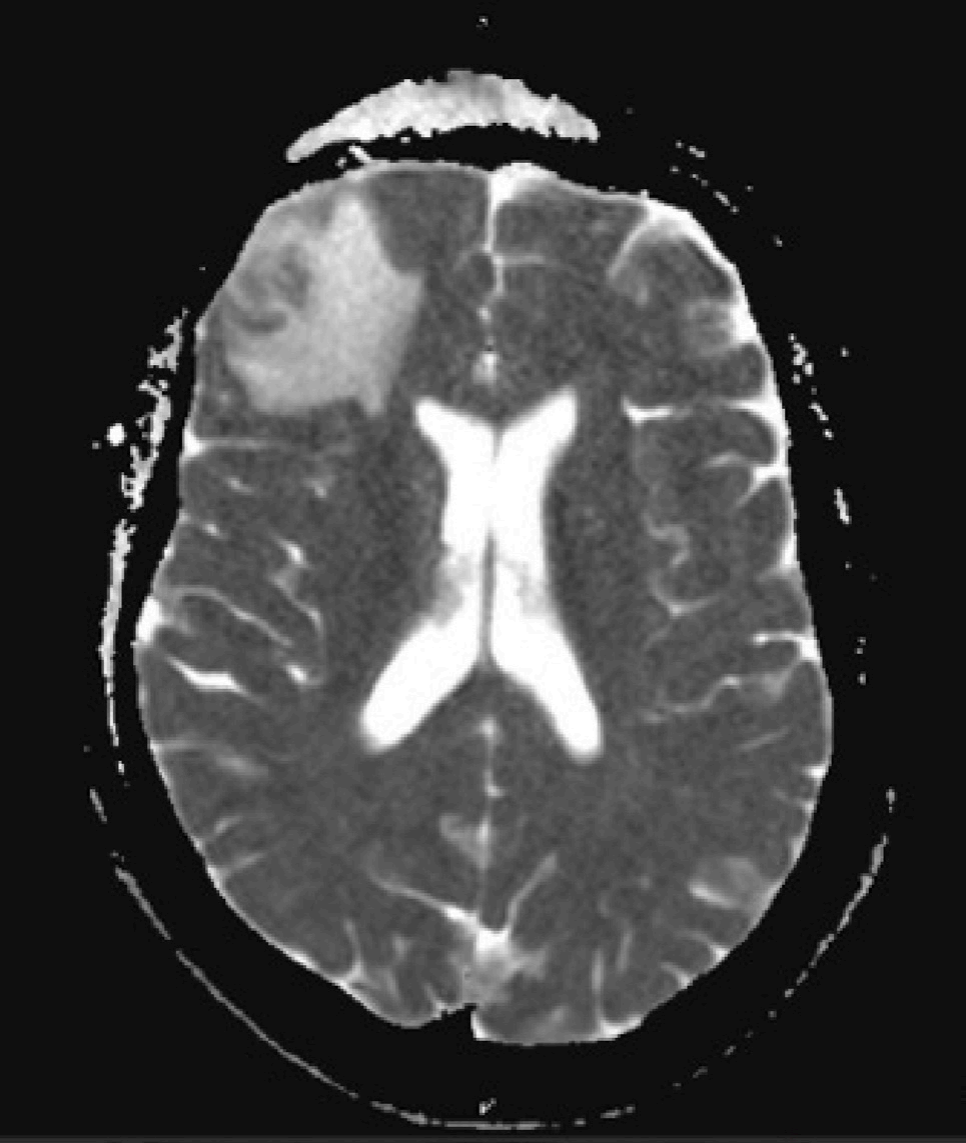

New solitary ring-enhancing lesion with significant surrounding vasogenic edema within the anterior right frontal lobe.

09/23/20 MRI Brain

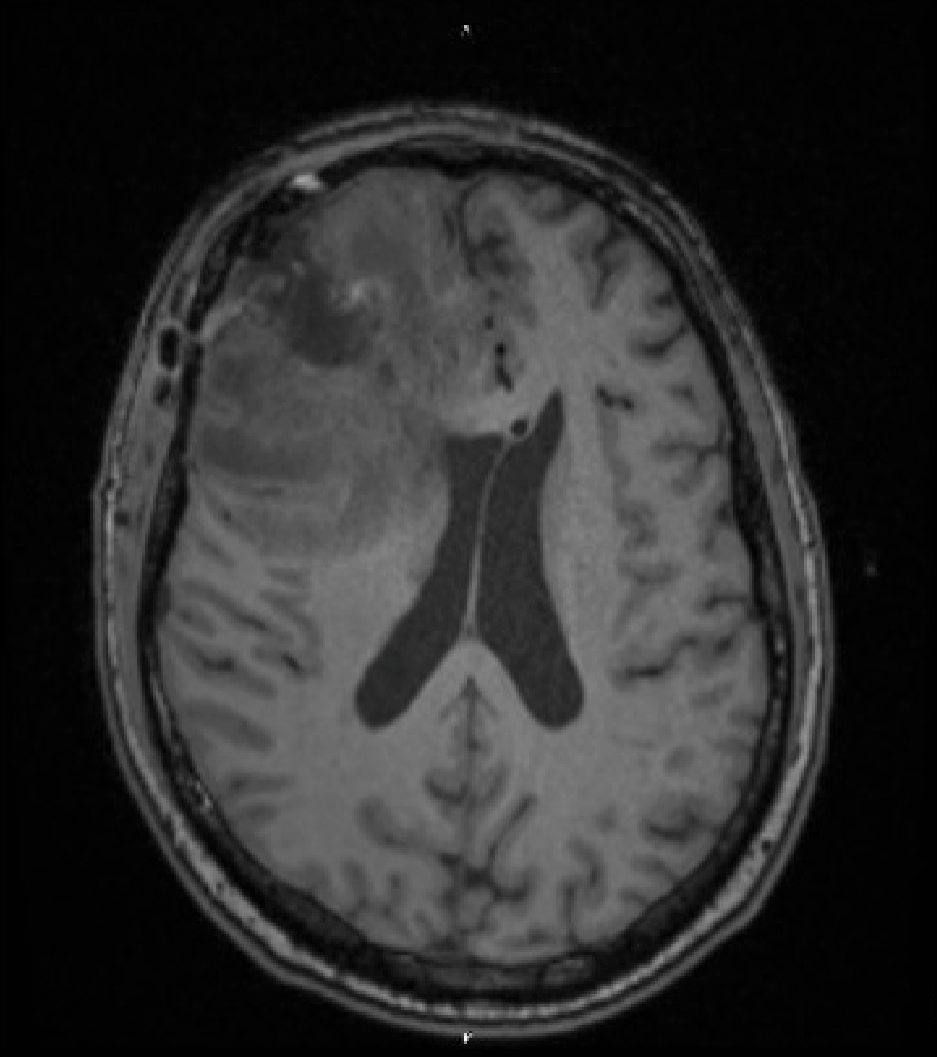

Post-Surgical right frontal lobe with edema, persistent cerebritis, and mass effect on the lateral ventricles.

Fluoribacter Bozemanae

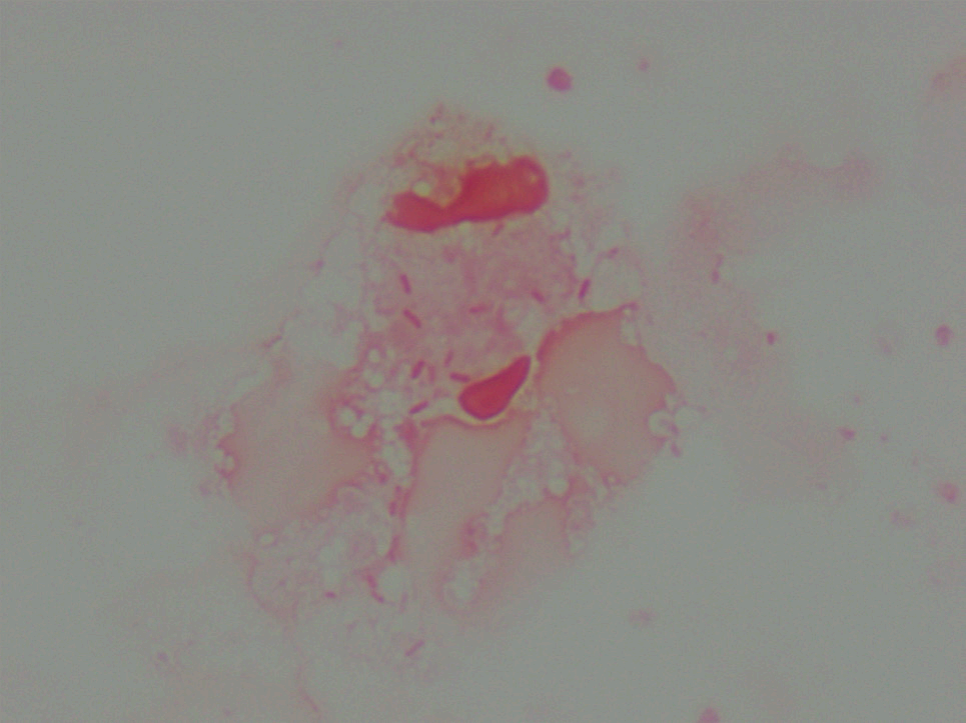

Formerly known as Legionella bozemanii, an intracellular GNR grown on BCYE.

**Conclusion:**

We present a diagnostically challenging case of *L. bozemanii* brain abscess in an immunocompromised patient. To our knowledge, this is the first case of culture proven *L. bozemanii* brain abscess in the literature. Considering the fastidious growth of the organism, fatal nature of the infection, and narrow therapeutic profile, *Legionella* infection should be considered in a multi-system disease in immunocompromised patients.

**Disclosures:**

**Wesley Kufel, PharmD**, **Melinta** (Grant/Research Support)**Merck** (Grant/Research Support)**Theratechnologies, Inc.** (Advisor or Review Panel member)

